# Rubella epidemic caused by genotype 1E rubella viruses in Beijing, China, in 2007–2011

**DOI:** 10.1186/1743-422X-10-122

**Published:** 2013-04-18

**Authors:** Meng Chen, Zhen Zhu, Donglei Liu, Guohong Huang, Fang Huang, Jiang Wu, Tiegang Zhang, Wenbo Xu, Xinghuo Pang

**Affiliations:** 1 Beijing Center for Diseases Prevention and Control, No. 16, Hepingli Middle Street, Dongcheng district, Beijing 100013, People’s Republic of China; 2 WHO WPRO Regional Reference Measles/Rubella Laboratory, Key Laboratory of Medical Virology Ministry of Health, National Institute for Viral Disease Control and Prevention, Chinese Center for Disease Control and Prevention, No.155, Changbai Road, Changping District, Beijing 102206, People’s Republic of China

**Keywords:** Rubella epidemics, Rubella virus, Genotype 1E, Beijing

## Abstract

**Background:**

A series of different rubella vaccination strategies were implemented to control rubella and prevent congenital rubella virus infection in Beijing, China. The rubella vaccine was available in 1995 in Beijing, and was introduced into the Beijing immunization program (vaccine recipients at their own expense vaccination) in 2000, and was introduced into the National Expanded Program on Immunization (vaccine recipients free vaccination) in 2006. Rubella virological surveillance started in Beijing in 2007.

**Results:**

The reported rubella incidence rate has decreased dramatically due to the introduction of the vaccine in Beijing since 1995. However, rubella epidemics occurred regardless in 2001 and 2007. The incidence rate among the floating population has gradually increased since 2002, reaching 2 or more times that in the permanent resident population. The peak age of rubella cases gradually changed from <15 years of age to adults after 2005. Phylogenetic analysis was performed and a phylogenetic tree was constructed based on the World Health Organization standard sequence window for rubella virus isolates. All Beijing rubella virus isolates belong to genotype 1E/cluster1 and were clustered interspersed with viruses from other provinces in China. The effective number of infections indicated by a Bayesian skyline plot remained constant from 2007 to 2011.

**Conclusions:**

The proportion of rubella cases among the floating population has increased significantly in Beijing since 2002, and the disease burden gradually shifted to the older age group (15- to 39-year olds), which has become a major group with rubella infection since 2006. Genotype 1E rubella virus continuously caused a rubella epidemic in Beijing in 2007–2011 and was the predominant virus, and all Beijing genotype 1E viruses belong to cluster 1, which is also widely circulated throughout the country.

## Background

Rubella infection usually presents as a mild or asymptomatic infection in children and adults. However, the rubella virus is a potent, highly infectious, and teratogenic agent that may cross the placenta and cause fetal infection; such infection in the first trimester of pregnancy can result in miscarriage, fetal death, or an infant born with serious birth defects including hearing impairment, cataracts, and cardiac defects, collectively known as congenital rubella syndrome (CRS) [1,2]. To prevent congenital rubella infection including CRS, the World Health Organization (WHO) recommended the introduction of a rubella-containing vaccine (RCV) in national childhood immunization schedules in 2000 [3]. Since then, the number of reported rubella cases has dramatically decreased worldwide [4].

The rubella virus is the single member of the *Rubivirus* genus in the Togaviridae family. Rubella virus has an enveloped, single-stranded, positive-polarity RNA genome consisting of 9,762 nucleotides (nt) that contains a 5′-proximal open reading frame (ORF) that encodes nonstructural polypeptides (p150 and p90) responsible for viral genome replication as well as a 3′-proximal ORF that encodes 3 structural polypeptides (C, E2, and E1) [1,5]. A 739-nucleotide region (nt8731–9469) within the *E1* gene was recommended as a standard genotyping window for assigning genotypes by comparison with the reference virus sequences [6]. Although rubella is a serologically monotypic virus [1], recent sequence analysis revealed that distinct genetic variants of rubella viruses exist [7]. To date, 2 clades (1 and 2), 9 genotypes (1B, 1C, 1D, 1E, 1 F, 1G, 2A, 2B, and 2C), and 4 provisional genotypes (1a, 1 h, 1i, and 1j) have been identified [7]. The genotype 1E and 2B viruses had wide geographic distribution and were frequently found in the world in recent years [8]. In addition, genotype 1E was the predominant genotype circulated in China since it was first found in 2001 [9,10].

Beijing, the capital city of China, covers an area of only 16,800 square kilometers and is composed of 14 districts and 2 counties and has a large population of more than 19.72 million individuals (data from Chinese Statistics Bureau in 2011), including more than 12.46 million permanent resident populations (the population of habitual residence in Beijing, including the provisional go out population) and approximately 7.26 million floating populations (the population of internal migration who leave the domicile and live in Beijing for the purpose of work or others). It is expected that the number of floating population in Beijing will continue to increase in the next 20 years.

In 1989, rubella including CRS was classified as a category C notifiable infectious disease by the Ministry of Health of China; rubella incidence data have been available in Beijing ever since. And in 2007, rubella virological surveillance started in Beijing started. In 2011, the CRS surveillance projects were initiated and sponsored by the Beijing Municipal Health Bureau in order to elucidate the disease burden of CRS, and in the CRS prevention stage, disease surveillance was focus on detecting cases of CRS.

To control rubella and prevent congenital rubella virus infection, a series of different rubella vaccination strategies were implemented in Beijing. In the first stage from 1995 to 1999, the rubella vaccine, including a domestic MMR vaccine containing rubella vaccine strain BRDII and an imported MMR vaccine containing rubella vaccine strain RA27/3, have been available in Beijing since 1995. In the second stage from 2000 to 2005, the rubella vaccine was introduced into the Beijing immunization program (vaccine recipients at their own expense vaccination), and the target population included 17-month-old children; 7-, 12-, and 16-year-old students; and new students in the university from outside of Beijing to cover all populations under 20 years of age in Beijing. In the third stage from 2006 to the present, a 2-dose schedule with the measles-mumps-rubella (MMR) vaccine was administrated to children at 1.5 years of age and 6 years of age, and the primary immunization dose was adjusted to 8 months of age in 2008 when the rubella vaccine was formally introduced to the national Expanded Program on Immunization (vaccine recipients free vaccination). That means all the children under 6 years of age have had the opportunity to be vaccinated (vaccinated three times at 1, 2 and 3 years of age) against the rubella virus free of cost since 2006.

Given that little is known about the rubella epidemic in Beijing, here, we provide an overview (from 1990 to date) of the rubella epidemiology in this city that has a high proportion of floating populations. In addition, although case-based rubella surveillance was integrated into the measles laboratory network in Beijing in 2005, rubella virological surveillance was started in 2007, and in this study, the circulation pattern of rubella viruses was investigated during the past 5 years to establish an important genetic baseline in Beijing during the rubella control stage.

## Results

### Overview of rubella epidemiology in Beijing

**Figure 1:**
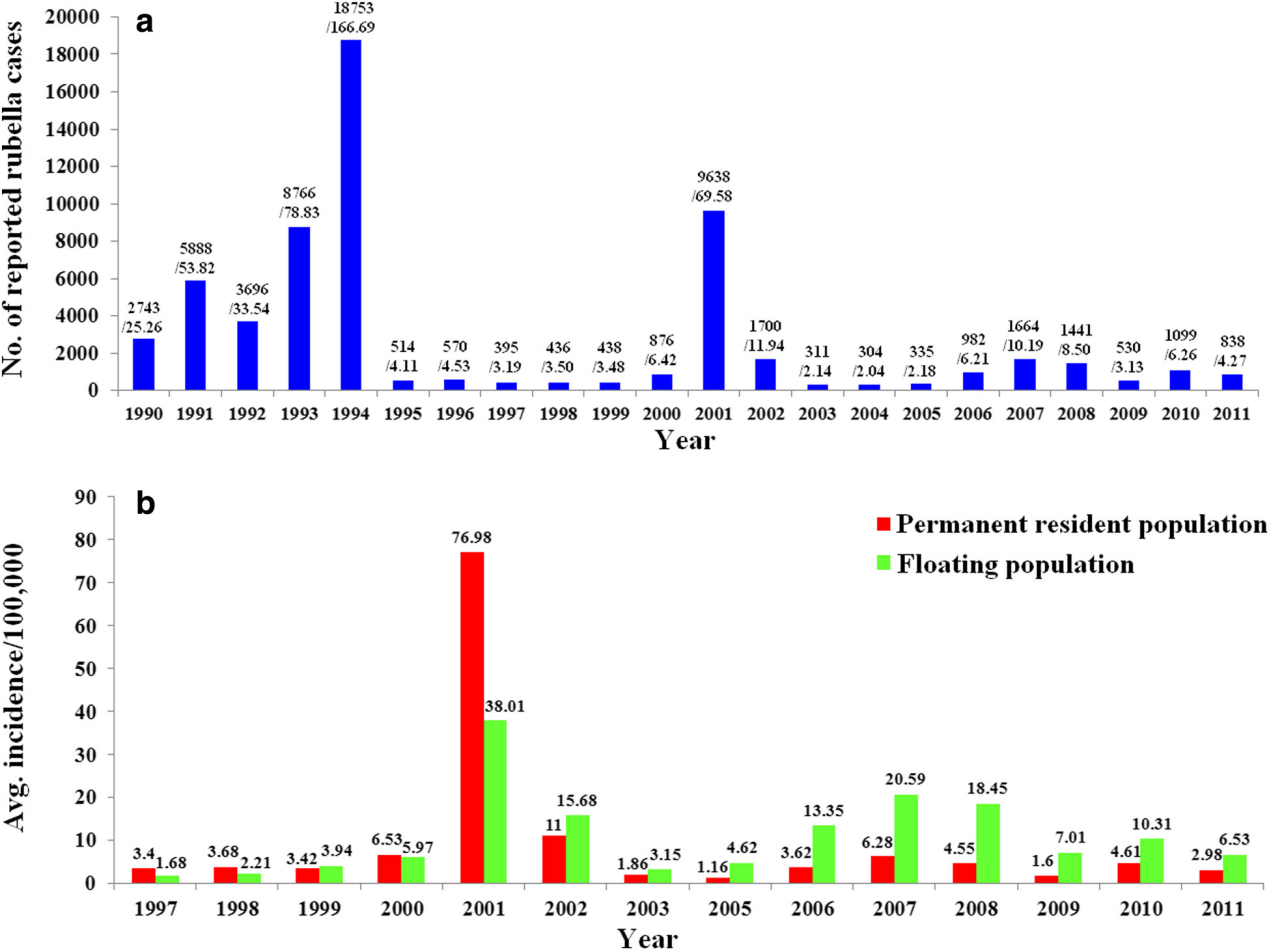
**Reported rubella cases in Beijing, China since 1990.** (**a**) Total rubella cases and the incidence rate in Beijing by year of onset, from 1999 to 2011. The numbers above the bars represented the number of reported rubella cases / the incidence rate. (**b**) Comparison of the rubella incidence rate between the permanent resident and floating populations by year of onset, from 1997 to 2011. Data pertaining to the rubella incidence rate of the permanent resident and floating populations in 2004 were not available.

During 1990–1994, the reported rubella incidence rate remained at a high level, peaking at 166.69/100,000 in 1994. Soon after, the reported incidence rate dramatically decreased along with the usage of the rubella vaccine in Beijing since 1995. However, rubella epidemics also occurred in 2001 (reported rubella cases: 9638; reported peak annual incidence rate, 69.58/100,000) and in 2007 (reported rubella cases: 1664; reported peak annual incidence rate, 10.19/100,000) (Figure 1a). Before 2001, the rubella incidence rate in the permanent resident population was almost the same or slightly higher than that in the floating population; on the contrary, the incidence rate among the floating population has gradually increased since 2002, reaching 2 or more times that of the permanent resident population (Figure 1b). Data pertaining to the rubella incidence rate of the permanent resident population and floating population in 2004 were not available.

**Figure 2:**
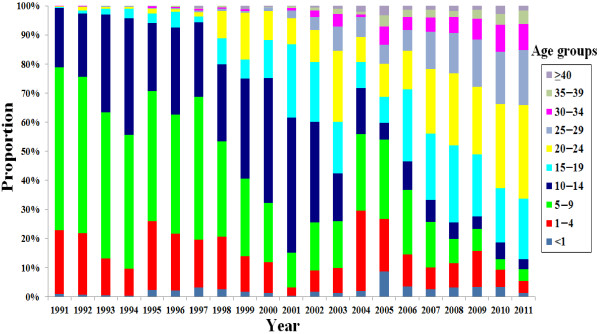
**Rubella incidences in different age groups in Beijing, China since 1991.** As reported by the case-based rubella surveillance system of the Beijing Center for Disease Prevention and Control.

The reported rubella cases were concentrated in patients <15 years of age before 2005 (>59.7%) except for 2003 (42.4%). However, the proportion of reported rubella cases within the 15- to 39-year-old age group increased each year from 1998 (20.2%) to 2011 (85.6%), and the 15- to 39-year-old age group became a major group with rubella infection between 2006 and 2011 (52.0%, 65.2%, 72.7%, 70.9%, 78.5%, and 85.6%, respectively, for each of these years) (Figure 2). The CRS surveillance project were initiated in order to elucidate the disease burden of CRS in 2011 in Beijing, and only 1 CRS case was captured and was confirmed by the laboratory through the surveillance system.

Rubella seroepidemiology analyses were performed in 2003 (2211 people including 1035 immigrants were recruited) and in 2007 (2003 people including 923 immigrants were recruited). The rates of sero-negativity in 2003 among permanent resident population and floating population were 24.4% and 30.8%, respectively. The sero-negativity of rubella antibodies for permanent resident population remained stable (25.6%), while it decreased to 28.0% for floating population in 2007.

### Genotype 1E rubella virus continuously caused a rubella epidemic in Beijing

**Figure 3:**
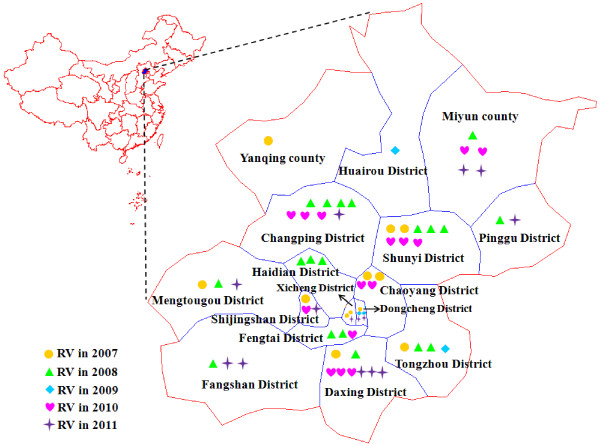
Geographic distribution of 64 genotype 1E rubella viruses isolated in Beijing, China, from 2007 to 2011.

**Figure 4:**
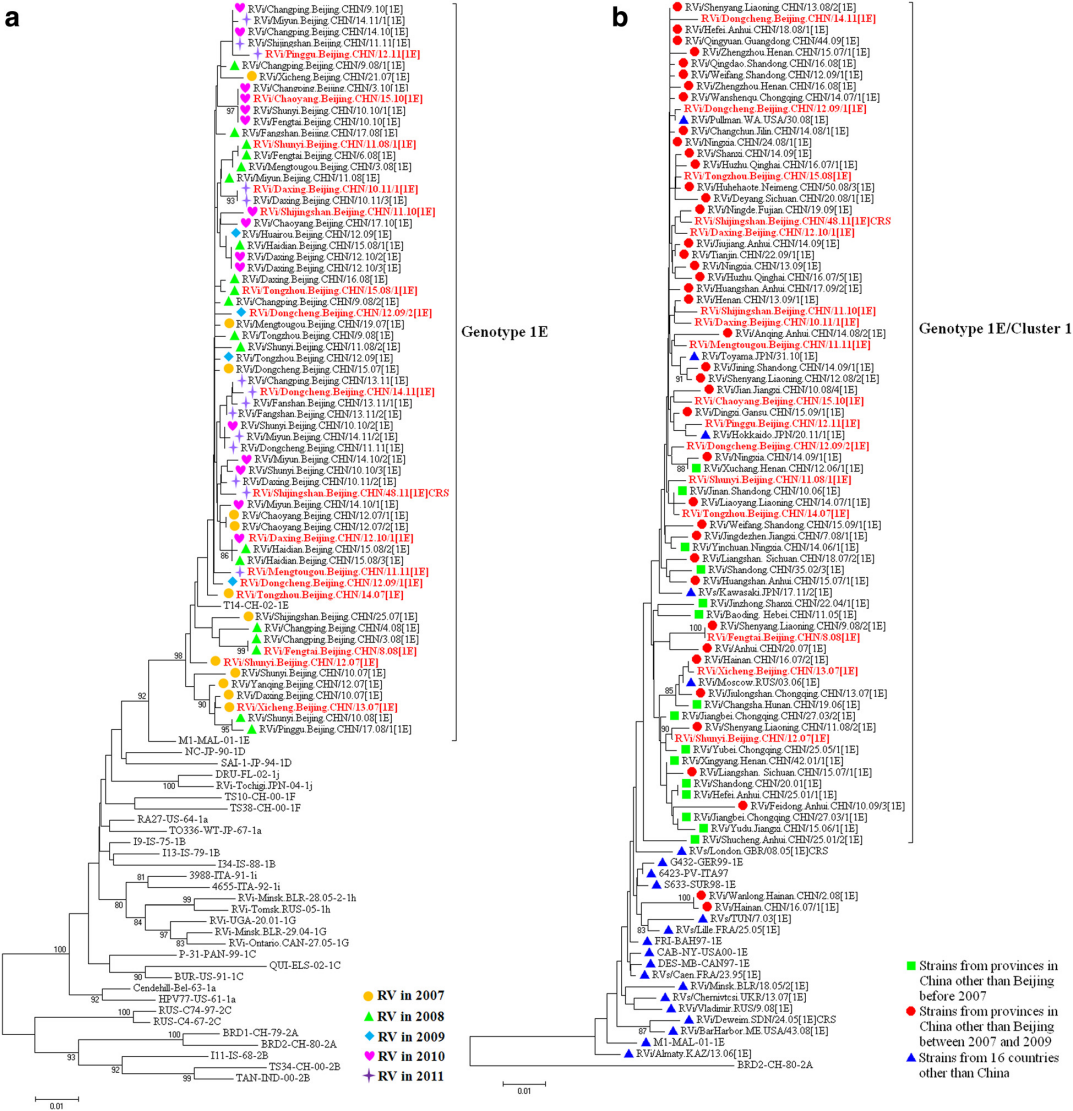
**Phylogenetic analyses of sequences of the Beijing rubella viruses based on the World Health Organization (WHO) standard sequence window.** (**a**) Phylogenetic analysis of 64 Beijing rubella virus sequences in 2007–2011 compared with 32 WHO reference sequences. The symbols with different colors represent rubella viruses isolated in different years. The 16 rubella viruses in red were selected for further molecular epidemiological analysis. (**b**) Phylogenetic analysis of 16 representative Beijing genotype 1E rubella virus strains compared with 57 genotype 1E rubella viruses from the other provinces of China in 2001–2009 and 22 viruses from 16 other countries. The genotype 1E rubella virus sequences other than Beijing were obtained from the GenBank database. The Chinese vaccine strain BRD II was used as an outgroup.

A total of 64 rubella viruses were isolated from throat swabs in all 16 districts/counties in 2007–2011, including 1 virus that was isolated from a lab-confirmed CRS case (Figure 3). All viruses were named according to WHO systematic nomenclature for rubella viruses. The sequences of the 739-nt region within the E1 gene of the 64 rubella virus isolates and 32 WHO reference strains were used for phylogenetic analysis (Figure 4a). All Beijing rubella virus isolates belong to genotype 1E (Figure 4a), which indicated that genotype 1E continuously caused a rubella epidemic in the past 5 years in Beijing. The nucleotide and amino acid sequence identity among these viruses was 97.2%–100% and 97.9%–100%, respectively. Multiple different clusters existed among Beijing rubella viruses in 2007–2008. Beijing rubella viruses isolated in 2009–2011 were quite similar (>98.5%) and had high similarity with some of the viruses during 2007–2008.

To determine the molecular epidemiology of the Beijing rubella virus strains, a phylogenetic dendrogram was constructed that contained 16 randomly selected Beijing rubella virus strains (3 strains in 2007, 3 strains in 2008, 2 strains in 2009, 3 strains in 2010, and 5 strains in 2011 selected) based on the their nucleotide divergence, lineage resolution and sampling time as indicated in Figure 4a, 57 strains from 19 provinces in mainland China in 2001–2009, and 22 strains from 16 other countries in 1995–2011 (Figure 4b and Table 1). Similar to the most rubella virus sequences from mainland China in 2001–2009, the Beijing rubella virus strains clearly belonged to genotype 1E/cluster1 and were interspersed with viruses from 19 provinces in China and from Russia (2006), the United States (2008), and Japan (2010 and 2011).

**Table 1:** Genotype 1E virus strains from 16 countries other than China used in this study

**Virus isolate**	**Isolation country and year**	**GenBank accession no.**	**Reference**
RVs/Caen.FRA/23.95/1E	France, 1995	FN546967	[11]
FRI-BAH97	Bahamas, 1997	AY326359	[12]
DES/MB-CAN97	Canada, 1997	AY326358	[12]
6423/PV-ITALY-1997	Italy, 1997	AY161374	[12]
S633-SUR98	Suriname, 1998	AY326363	[12]
G432-GER99	Germany, 1999	AF551761	[12]
CAB/NY-USA00	USA, 2000	AY326355	[12]
M1-MAL-2001	Malaysia, 2001	AY968211	[6]
RVs/TUN/7.03[1E]	Tunisia, 2003	FN547014	[11]
Rvi/Deweim.SDN/24.05[1E]CRS	Sudan, 2005	FJ775000	[13]
RVi/Minsk.BLR/18.05/2[1E]	Belarus, 2005	AM258955	[14]
RVs/London.GBR/08.05[1E]CRS	UK, 2005	EF210051	[15]
RVs/Lille.FRA/25.05[1E]	France, 2005	FN547019	[11]
RVi/Moscow.RUS/03.06[1E]	Russia, 2006	FJ711682	
RVi/Almaty.KAZ/13.06[1E]	Kazakhstan, 2006	FJ711684	
RVs/Chernivtcsi.UKR/13.07[1E]	Ukraine, 2007	FJ711683	
RVi/Pullman.WA.USA/30.08[1E]	USA, 2008	JN635288	
RVi/BarHarbor.ME.USA/43.08[1E]	USA, 2008	JN635286	
RVi/Vladimir.RUS/9.08[1E]	Russia, 2008	FJ711681	
RVi/Toyama.JPN/31.10[1E]	Japan, 2010	AB646368	
RVs/Kawasaki.JPN/17.11/2[1E]	Japan, 2011	AB674471	
RVi/Hokkaido.JPN/20.11/1[1E]	Japan, 2011	AB683468	

**Figure 5:**
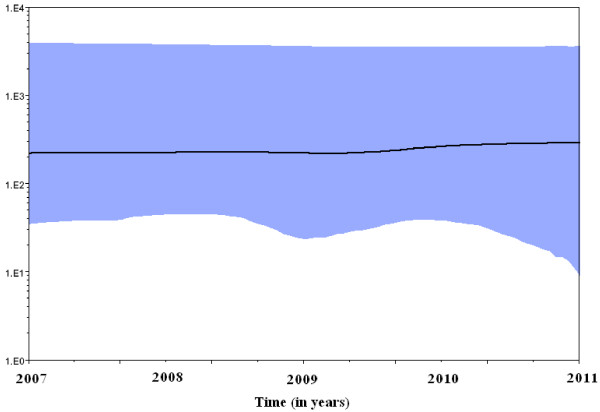
**Bayesian skyline plot obtained by analyzing the 16 genotype 1E rubella virus sequences.** Ordinate: the number of effective infections at a given time; abscissa: time (in years). The thick solid line represents the median, while the blue area represents the 95% highest posterior density of the number of effective infections at the time estimates.

Both the strict and relaxed clock models were implemented using the Bayesian skyline model for population growth, the latter being preferred. A Bayesian skyline plot (BSP; Figure 5) showed that the effective number of infections remained constant in 2007–2011.

## Discussion

An overview of the rubella epidemiology in Beijing was provided in this study. The rubella epidemic cycles occurred approximately every 6–7 years (1994, 2001, and 2007), a trend that was similar to the national trend [10]. With the introduction of the rubella vaccine in 1995, a significant reduction in rubella morbidity has occurred, and the incidence rate has remained at a relatively low level. However, the proportion of rubella cases among the floating population has increased significantly since 2002, which may be due to high migration and tourism rates over the past 10 years. In addition, seroepidemiological surveys in 2007 estimated that susceptibility to rubella among the floating population was 28%; therefore, as the size of the floating population increases in the near future, it is necessary to take effective measures to improve RCV coverage among these people, otherwise rubella control in Beijing may be impeded.

Reported rubella cases were concentrated in patients < 5 years of age before 2005, while the disease burden gradually shifted to the older age group (15- to 39-year olds), which has remained a major group with rubella infection since 2006. The occurrence of this phenomenon may due to the low vaccine coverage in Beijing at that time. Low coverage might alter the rubella virus transmission dynamics and could increase the average age of rubella infection for females from childhood to the childbearing age group, meaning that the risk for CRS might increase [4]. Such a shift in susceptibility of older age groups occurred and was documented in Greece and South Africa as well [16,17]. Therefore, following the WHO recommendations, regions or countries attempting rubella control and elimination should ensure that routine vaccine coverage in children is >85% and sustainable in the long term to ensure rubella immunity among women of reproductive age [3,18].

Since the rubella incidence rate has become much higher among the childbearing age group in recent years in Beijing, the disease burden of CRS needed to be elucidated; thus, CRS surveillance projects were initiated and sponsored by the Beijing Municipal Health Bureau in 2011. To date, only 1 CRS case was captured through the surveillance system. Birth defects including a cataract in the left eye and rubella-specific IgM antibody were detected after birth in this individual. Phylogenetic analysis indicated that the rubella virus of predominant genotype (genotype 1E) caused the congenital infections. Although CRS surveillance is challenging because of the difficulty of diagnosis and reporting in settings with limited medical resources, ongoing CRS surveillance is necessary. A comprehensive and high-quality program including CRS surveillance, integrated rubella surveillance in measles case-based surveillance, and a well-functioning routine immunization program are needed to fully understand the burden of rubella and CRS [4] and determine the most appropriate strategies for rubella control and CRS prevention in Beijing. In addition, effective routine rubella and CRS surveillance has not yet been established in China; therefore, a successful experience with rubella and CRS surveillance in Beijing could be extended to the entire country in the future.

The WHO Global Measles and Rubella Laboratory Network was developed in 2000 and currently includes 690 laboratories serving 183 countries including China [19]. An important aspect of laboratory surveillance for measles and rubella is the genetic characterization of circulating wild-type viruses to support molecular epidemiologic studies [19]. In Beijing, rubella virological surveillance was integrated into the measles surveillance in 2007, and 5-year consecutive surveillance indicated that the predominant genotype 1E rubella virus continuously caused a rubella epidemic in Beijing in the past 5 years. Between 2007 and 2008, multiple transmission chains of genotype 1E rubella viruses were found in different districts/counties of Beijing, while some transmission chains of genotype 1E faded, and viruses with similar sequences continued circulating in various districts/counties in the following 3 years. This might be related to the large-scale use of the rubella vaccine since 2006, when the rubella vaccination was provided free of charge to all children in Beijing. Vaccination may have interrupted some transmission chains of the rubella virus, while some genotype 1E strains during 2007–2008 survived and continued circulating in 2009–2011.

Effective population size determines the strength of genetic drift and the frequency of co-infection by multiple genotypes, making it a key factor in viral evolution. Viral evolutionary dynamics on different spatiotemporal scales will be largely determined and constrained by effective population size. For viruses, effective population size can be considered as the average number of horizontally transmitted virions in an idealized population, and it will determine how strong genetic drift – one of the key evolutionary forces – acts on viral populations [20,21].

To estimate virus effective population sizes through time, we used a coalescent -based approach that can independently estimate effective population sizes, and this approach is implemented in a Bayesian Markov Chain Monte Carlo (MCMC) inference framework in the program Bayesian evolutionary analysis sampling trees (BEAST) [22]. The phylogeny and divergence times of the genotype 1E rubella virus lineages using a standard sequence window sampled at different times were inferred using a relaxed molecular clock model in non-parametric BSP method, which is a useful analysis based on coalescent theory seeks to predict the amount of time elapsed between the introduction of a mutation and the arising of a particular gene distribution in a population. Effective population size can be defined as the census population size divided by the variance in the number of offspring among individuals. In the case of a virus, the census population size is estimated from isolate counts, whereas the effective population size is actually an estimate of the effective number of infections, that is, those that go on to produce subsequent infections [23,24].

The results showed that the rubella virus population size in Beijing remained constant in 2007–2011, although a free 2-dose schedule with MMR was administrated in 2006 and a free 3-dose schedule with MMR was administrated since 2008. The results indicated that genotype 1E rubella viruses have constantly circulated since 2007 and are the predominant genotype in Beijing. Compared with the situation of the rubella virus population size nationwide, the epidemic started a decline that led to a decrease in the effective population size along with the introduction of rubella vaccine into the National Expanded Program on Immunization [10], this is obviously different and may be due to the migration to Beijing from other provinces, resulting in a high proportion of floating population, among which the rubella vaccine coverage may be much lower.

Chinese genotype 1E viruses could be divided into 2 clusters. Similar to the findings of an earlier report [10], all Beijing rubella virus strains belong to genotype 1E/cluster1 and are interspersed with viruses from other provinces of China. Furthermore, all genotype 1E/cluster1 rubella viruses circulated in different provinces in mainland China were chronologically well correlated. This finding suggests that the rubella virus did not evolve independently in Beijing. Instead, these strains co-evolved and co-circulated with those from other provinces in China. Recent studies suggest a very wide circulation of genotype 1E [8], which may be the most prevalent contemporary genotype worldwide [8], while genotype 1E/cluster 1 is unique to China [10]. This study suggests that the genotype 1E/cluster1 was successively imported into Russia (2006), United States (2008), and Japan (2010 and 2011), a phenomenon that may be related with the frequent international exchanges.

Although the important genetic baseline of Beijing has already been established, gaps for rubella virological surveillance still exist; therefore, ongoing molecular epidemiological surveillance of circulating viruses is needed, especially when routine vaccination programs are performed. Vaccination will gradually decrease or eliminate endemic viruses and allow for the detection of imported viruses with multiple genotypes.

## Methods

### Rubella epidemiology data

Data on the number of reported rubella cases and the annual rubella incidence rates in 1990–2004 were taken directly from the infectious diseases system of Beijing and from the case-based rubella surveillance system of Beijing since 2005 due to rubella cases reporting was introduced into the National Notifiable Disease Reporting System (NNDRS), and it was the official source of data on number of reported rubella cases since then. The surveillance system covers all the level of Center for Disease Prevention and Control (CDC) and medical institutions in Beijing. The case was reported by clinicians through the real time online reporting system.

Any patient presents with fever, maculopapular rash, and cervical, suboccipital or postauricular adenopathy or arthralgia/arthritis was considered as a suspected rubella case, and when the suspected rubella case with a positive blood test for rubella-specific Immunoglobulin M (IgM), it becomes a laboratory-confirmed rubella case. Any infant less than one year of age with a qualified physician detects presents at least two of the complications listed in (a) below or one in (a) and one in (b) was considered as a clinically confirmed CRS case: (a) Cataract, congenital glaucoma, congenital heart disease, loss of hearing, pigmentary retinopathy; (b) Purpura, splenomegaly, microcephaly, mental retardation, meningocephalitis, radiolucent bone disease, jaundice that begins within 24 hours after birth, and when a clinically-confirmed CRS case who has a positive blood test for rubella-specific IgM, it becomes laboratory confirmed CRS case.

### Specimen collection, virus isolation, and identification

This study did not involve human participants or human experimentation; the only human materials used were throat swab samples collected from suspected rubella patients at the instigation of the Ministry of Health P. R. of China for public health purposes, and written informed consent for the use of their clinical samples was obtained from all patients involved in this study. This study was approved by the second session of the Ethics Review Committee of the National Institute for viral disease control and prevention, Chinese Center for Disease Control and Prevention. Throat swab samples were collected from suspected rubella patients within 3 days after rash onset during the case-based rubella surveillance in 2007–2011. In addition, a throat swab from a lab-confirmed CRS case in 2011 was also included in this study.

Isolation of the rubella virus was performed using African green monkey kidney cells transfected with signaling lymphocytic activation molecule (Vero/Slam) following the standard protocol [25]. The presence of viral RNA was detected using real-time reverse transcription-polymerase chain reaction (RT-PCR) after RNA extraction from infected tissue culture cells by using a QIAamp Viral RNA Extraction Mini Kit (Qiagen, Valencia, CA). The real-time RT-PCR was performed using 185-bp amplicon primers and the TaqMan probe as previously described with a One Step PrimeScript® RT-PCR Kit (Perfect Real Time; TaKaRa Biotechnology [Dalian] Co., Ltd.) [26]. The assay was performed with 25-μL reaction mixtures containing reaction buffer, 12.5 μL of 2× One Step RT-PCR Buffer III, 2.5 U of TaKaRa Ex Taq HS, 0.5 μL of PrimeScript RT Enzyme Mix II, 0.5 μM each primer, 0.1 μM Taqman probe, and 5 μL of RNA. The thermal cycling was carried out using an ABI 7500 Thermal Cycler with 42°C for 30 min, 95°C for 2 min, 40 cycles of 95°C for 15 s, and 60°C for 1 min. A reaction mixture containing water as the template was run on each plate as a negative control, and positive controls were also performed. The data were analyzed with 7500 software (version 2.0.1), and the signal for all wells in cycles 3–15 was used as the background signal.

### WHO standard genotyping window amplification

RT-PCR was performed by using the primer pair from US CDC (forward primer: 5^′^-AGCGACGCGGCCTGCTGGGG-3^′^; reverse primer: 5^′^-CGCCCAGGTCTGCCGGGTCTC-3^′^) to amplify a 944-nt (nt 8633–9577) product containing the 739-nt genotyping window with a One Step RNA PCR (AMV) Kit (TaKaRa Biotechnology [Dalian] Co., Ltd.). RT-PCR reaction mixture preparation and thermal cycling was done following the kit instructions. DNA from RT-PCR-positive reactions was purified using a QIA Gel Extraction Kit (Qiagen Valencia, CA) and then determined bidirectionally using the dye terminator method (BigDye Terminator Version 3.1 Cycle Sequencing Kit; Applied Biosystems, Foster City, CA, USA) and an ABI 3100 Genetic Analyzer (Applied Biosystems, Hitachi, Tokyo, Japan). Sequence data were edited and assembled with Sequencher software (Version 5.0; GeneCode, Annarbor, MI) to obtain the 739-nt sequence window.

### Phylogenetic analysis and the demographic history estimation

Alignment of the 739-nt sequences of the rubella virus isolates was performed using BioEdit Sequence Alignment Editor Software (version 7.0.9; Tom Hall, North Carolina State University, Raleigh, NC, USA). Phylogenetic trees were constructed using the neighbor-joining Kimura 2-parameter distance method using the MEGA program (version 5.0; Sudhir Kumar, Arizona State University, Tempe, AZ, USA) [27]. Bootstrap values > 80% were considered statistically significant for grouping.

In order to predict the amount of time elapsed between the introduction of a mutation and the arising of a particular gene distribution in a population, a BSP, a retrospective model of population genetics, was used under both strict and relaxed (uncorrelated lognormal distributed) clock conditions to estimate the demographic history with the 739-nt sequence window of the rubella viruses isolated in Beijing, which allows for estimates of effective population size over time with credibility intervals at every time depending on errors due to the phylogeny reconstruction and the stochastic nature of the coalescent process [28].

### Nucleotide sequence accession numbers

The nucleotide sequences of 64 rubella virus strains isolated in this study have been deposited in the GenBank database under accession numbers JQ979489–JQ979552. An additional 57 sequences for rubella viruses from 19 provinces other than Beijing in mainland China were retrieved from the GenBank database.

## Abbreviations

BSP: Bayesian skyline plot; CDC: Center for disease control and prevention; CRS: Congenital rubella syndrome; MMR: Measles-Mumps-Rubella; ORF: Open reading frame; PCR: Polymerase chain reaction; RCV: Rubella-containing vaccine; WHO: World health organization.

## Competing interests

The authors declare that they have no competing interests.

## Authors’ contributions

MC, ZZ, DLL, WBX, and XHP prepared the manuscript. FH, JW, WBX, and XHP designed the study and organized the coordination. MC and TGZ collected the specimens and performed the virus isolation and identification processes. MC, ZZ, DLL, and GHH performed the data analysis. All authors read and approved the final manuscript.

## References

[B1] Frey TK Molecular biology of rubella virus Adv Virus Res 1994 44 69 160 7817880 10.1016/S0065-3527(08)60328-0 PMC7131582

[B2] Lee JY Bowden DS Rubella virus replication and links to teratogenicity Clin Microbiol Rev 2000 13 571 587 10.1128/CMR.13.4.571-587.2000 11023958 PMC88950

[B3] World Health Organization Rubella vaccine. WHO position paper Wkly Epidemiol Rec 2000 75 161 172

[B4] Reef SE Strebel P Dabbagh A Gacic-Dobo M Cochi S Progress toward control of rubella and prevention of congenital rubella syndrome–worldwide, 2009 J Infect Dis 2011 204 Suppl 1 S24 S27 21666168 10.1093/infdis/jir155

[B5] Chen M Icenogle JP Banatvala J, Peckham C Molecular virology of rubella virus, p1-18 Rubella virus 2007 Oxford, United Kindom: Elsevier

[B6] World Health Organization Standardization of the nomenclature for genetic characteristics of wild-type rubella viruses Wkly Epidemiol Rec 2005 80 126 132 15850226

[B7] World Health Organization Global distribution of measles and rubella genotypes--update Wkly Epidemiol Rec 2006 81 474 479 17175602

[B8] Abernathy ES Hubschen JM Muller CP Jin L Brown D Komase K Mori Y Xu W Zhu Z Siqueira MM Status of global virologic surveillance for rubella viruses J Infect Dis 2011 204 Suppl 1 S524 S532 21666209 10.1093/infdis/jir099

[B9] Zhu Z Abernathy E Cui A Zhang Y Zhou S Zhang Z Wang C Wang T Ling H Zhao C Rubella virus genotypes in the People's Republic of China between 1979 and 2007: a shift in endemic viruses during the 2001 Rubella Epidemic J Clin Microbiol 2010 48 1775 1781 10.1128/JCM.02055-09 20351211 PMC2863877

[B10] Zhu Z Cui A Wang H Zhang Y Liu C Wang C Zhou S Chen X Zhang Z Feng D Emergence and continuous evolution of genotype 1E rubella viruses in China J Clin Microbiol 2012 50 353 363 10.1128/JCM.01264-11 22162559 PMC3264136

[B11] Vauloup-Fellous C Hubschen JM Abernathy ES Icenogle J Gaidot N Dubreuil P Parent-du-Chatelet I Grangeot-Keros L Muller CP Phylogenetic analysis of rubella viruses involved in congenital rubella infections in France between 1995 and 2009 J Clin Microbiol 2010 48 2530 2535 10.1128/JCM.00181-10 20463161 PMC2897492

[B12] Zheng DP Frey TK Icenogle J Katow S Abernathy ES Song KJ Xu WB Yarulin V Desjatskova RG Aboudy Y Global distribution of rubella virus genotypes Emerg Infect Dis 2003 9 1523 1530 14720390 10.3201/eid0912.030242 PMC3034328

[B13] Omer A Abdel Rahim EH Ali EE Jin L Primary investigation of 31 infants with suspected congenital rubella syndrome in Sudan Clin Microbiol Infect 2010 16 678 682 10.1111/j.1469-0691.2009.02966.x 19732080

[B14] Hubschen JM Yermalovich M Semeiko G Samoilovich E Blatun E De Landtsheer S Muller CP Co-circulation of multiple rubella virus strains in Belarus forming novel genetic groups within clade 1 J Gen Virol 2007 88 1960 1966 10.1099/vir.0.82580-0 17554029

[B15] Jin L Thomas B Application of molecular and serological assays to case based investigations of rubella and congenital rubella syndrome J Med Virol 2007 79 1017 1024 10.1002/jmv.20847 17516526

[B16] Panagiotopoulos T Antoniadou I Valassi-Adam E Increase in congenital rubella occurrence after immunisation in Greece: retrospective survey and systematic review BMJ 1999 319 1462 1467 10.1136/bmj.319.7223.1462 10582926 PMC28289

[B17] Schoub BD Harris BN McAnerney J Blumberg L Rubella in South Africa: An impending Greek tragedy? S Afr Med J 2009 99 515 519

[B18] World Health Organization Rubella vaccines: WHO position paper–recommendations Vaccine 2011 29 8767 8768 10.1016/j.vaccine.2011.08.061 21930175

[B19] Featherstone DA Rota PA Icenogle J Mulders MN Jee Y Ahmed H de Filippis AM Ramamurty N Gavrilin E Byabamazima C Expansion of the global measles and rubella laboratory network 2005–09 J Infect Dis 2011 204 Suppl 1 S491 S498 21666205 10.1093/infdis/jir107

[B20] Moya A Elena SF Bracho A Miralles R Barrio E The evolution of RNA viruses: A population genetics view Proc Natl Acad Sci U S A 2000 97 6967 6973 10.1073/pnas.97.13.6967 10860958 PMC34371

[B21] Zhang Y Wang J Guo W Wang H Zhu S Wang D Bai R Li X Yan D Zhu Z Emergence and transmission pathways of rapidly evolving levolutionary branch C4a strains of human enterovirus 71 in the central plain of China PLoS One 2011 6 e27895 10.1371/journal.pone.0027895 22125635 PMC3220707

[B22] Drummond AJ Rambaut A BEAST: Bayesian evolutionary analysis by sampling trees BMC Evol Biol 2007 7 214 10.1186/1471-2148-7-214 17996036 PMC2247476

[B23] Bennett SN Drummond AJ Kapan DD Suchard MA Munoz-Jordan JL Pybus OG Holmes EC Gubler DJ Epidemic dynamics revealed in dengue evolution Mol Biol Evol 2010 27 811 818 10.1093/molbev/msp285 19965886 PMC2877535

[B24] Wang C Zhu Z Xu Q Xu A Fang X Song L Li W Xiong P Xu W Rubella epidemics and genotypic distribution of the rubella virus in Shandong Province, China, in 1999–2010 PLoS One 2012 7 e42013 10.1371/journal.pone.0042013 22911874 PMC3404038

[B25] Zhu Z Xu W Abernathy ES Chen MH Zheng Q Wang T Zhang Z Li C Wang C He W Comparison of four methods using throat swabs to confirm rubella virus infection J Clin Microbiol 2007 45 2847 2852 10.1128/JCM.00289-07 17596370 PMC2045274

[B26] Abernathy E Cabezas C Sun H Zheng Q Chen MH Castillo-Solorzano C Ortiz AC Osores F Oliveira L Whittembury A Confirmation of rubella within 4 days of rash onset: comparison of rubella virus RNA detection in oral fluid with immunoglobulin M detection in serum or oral fluid J Clin Microbiol 2009 47 182 188 10.1128/JCM.01231-08 19005151 PMC2620850

[B27] Tamura K Dudley J Nei M Kumar S MEGA4: Molecular Evolutionary Genetics Analysis (MEGA) software version 4.0 Mol Biol Evol 2007 24 1596 1599 10.1093/molbev/msm092 17488738

[B28] Drummond AJ Rambaut A Shapiro B Pybus OG Bayesian coalescent inference of past population dynamics from molecular sequences Mol Biol Evol 2005 22 1185 1192 10.1093/molbev/msi103 15703244

